# *Porphyromonas gingivalis*-Induced NLRP3 Inflammasome Activation and Its Downstream Interleukin-1β Release Depend on Caspase-4

**DOI:** 10.3389/fmicb.2020.01881

**Published:** 2020-08-13

**Authors:** Pei-Hui Ding, Meng-Xin Yang, Na-Na Wang, Li-Jian Jin, Yan Dong, Xia Cai, Li-Li Chen

**Affiliations:** ^1^Department of Periodontology, Stomatology Hospital of Zhejiang University School of Medicine, Hangzhou, China; ^2^Key Laboratory of Oral Biomedical Research of Zhejiang Province, Zhejiang University School of Stomatology, Hangzhou, China; ^3^Department of Periodontology, Second Affiliated Hospital, School of Medicine, Zhejiang University, Hangzhou, China; ^4^Department of Periodontology, Fuyang People’s Hospital, Fuyang, China; ^5^Division of Periodontology and Implant Dentistry, Faculty of Dentistry, The University of Hong Kong, Hong Kong, China; ^6^Department of Prosthodontics, The Second Affiliated Hospital of Zhejiang University School of Medicine, Hangzhou, China

**Keywords:** NLRP3 inflammasome, Caspase-1, Caspase-11, pathogenic bacteria, commensal bacteria, adenosine 5'-triphosphate

## Abstract

**Background**: Oral commensals contribute to microbe-host symbiosis in periodontal homeostasis, and *Porphyromonas gingivalis* (*P. gingivalis*) as the keystone pathogen critically accounts for the shift of symbiosis to dysbiosis and periodontal destruction. Nucleotide-binding oligomerization domain (NOD)-like receptor (NLR) family pyrin domain containing 3 (NLRP3) inflammasome-mediated interleukin-1β (IL-1β) is significantly involved in periodontal diseases, and notably *P. gingivalis* enables to modulate the induction and expression of NLRP3. Whereas, the exact mechanism by which NLRP3 inflammasome is regulated in response to commensal and pathogenic bacteria remains unclear.

**Methods**: To examine the expression of IL-1β and NLRPs inflammasome in tissues with severe chronic periodontitis, and further investigate how Caspase-4-dependent non-canonical NLRP3 inflammasome pathways functioned during the interactions of *Streptococcus mitis* (*S. mitis*) and *P. gingivalis* with human THP-1 cells.

**Results**: IL-1β and NLRP3, NLRP6, NLRP12, and absent in melanoma 2 (AIM2) inflammasomes are highly expressed in gingival tissues with severe chronic periodontitis. In human THP-1 cells, *P. gingivalis* activates the synthesis and secretion of IL-1β to higher levels than *S. mitis*. Importantly, NLRP3-, Caspase-1-, and Caspase-4-siRNA knockdown THP-1 cells treated with *P. gingivalis* exhibited a lower expression level of IL-1β as compared to the control cells. In addition, silencing of either CASP4 or CASP1 can lead to a concurrent or reciprocal decrease in the expression of the other. Of note, the IL-1β induction is not affected in the *S. mitis*-treated THP-1 cells with the silence of NLRP3, Caspase-1, and Caspase-4 genes.

**Conclusion**: NLRP3/Caspase-4 and NLRP3/Caspase-1 dependent IL-1β production may crucially contribute to the dysregulated immuno-inflammatory response in periodontal pathogenesis.

## Introduction

The means by which the human immune system discriminates between commensal and pathogenic bacteria is particularly important in periodontal tissues. Trillions of commensal microorganisms continually interact with the immune system without eliciting a pro-inflammatory response (Lebeer et al., 2010), whereas keystone pathogens like *Porphyromonas gingivalis* (*P. gingivalis*) result in dysbiosis of periodontium during the process of chronic periodontitis (Hajishengallis et al., 2011). Furthermore, human host defenses allow colonization by probiotics, while pathogenic bacteria elicit an immune system clearance response (Janeway, 1992; Roberts and Darveau, 2002; La Fata et al., 2018). Specifically, inflammasomes, proteins belonging to the nucleotide-binding oligomerization domain (NOD)-like receptor (NLR) family, are intracellular pattern recognition receptors (PRRs) that are activated upon recognition of invasion by microbes (Kawai and Akira, 2011). Among them, the NLR family pyrin domain containing 3 (NLRP3) inflammasome is the most complex and responds to a large number of ligands, including microorganisms, microbial toxins, and intracellular risk signals. The pyrin domain (PYD), located in the N terminus of NLRP3, can recruit apoptosis-related point-like protein (ASC), followed by the precursor of Caspase-1 to form the NLRP3 inflammasome complex (Franchi et al., 2009). The assembly and activation of NLRP3 inflammasomes lead to enhanced secretion of mature interleukin-1β (IL-1β) and IL-18, through targeted cleavage by Caspase-1.

Bostanci et al. (2009) discovered that gene expression of NLRP3 in gingival tissues was significantly higher in patients with periodontal disease in comparison to healthy tissues, and was positively correlated with expression of IL-1β, in consistent with the study conducted by Xue et al. (2015). Xue further found that increased NLRP3 protein expression in gingival tissues with periodontitis. Moreover, supragingival and subgingival plaque biofilms were revealed to contribute to regulation of NLRP3 expression. For example, supernatant of the latter was found to down-regulate NLRP3 inflammasomes and IL-1β. Importantly, the removal of *P. gingivalis* from subgingival plaque biofilms leads to restored expression of NLRP3 and IL-1β (Bostanci et al., 2011). The above studies demonstrated that NLRP3 inflammasomes may have differential responses to the presence of individual bacterial taxa, and in particular *P. gingivalis* provided a strong signal in the suppression of NLRP3.

Other studies have also demonstrated that *P. gingivalis* inhibited NLRP3 activation (Taxman et al., 2012; Huck et al., 2015). In addition, *P. gingivalis* fimbriae inhibited the P2X7-dependent induction of IL-1β secretion by extracellular adenosine 5′-triphosphate (eATP) in mouse bone marrow macrophages (Morandini et al., 2014). Yilmaz et al. (2010) showed that *P. gingivalis* was not able to induce secretion of IL-1β unless exogenous ATP was also added to activate NLRP3 inflammasomes in gingival epithelial cells. In contrast, some reports have suggested a triggering effect by *P. gingivalis* on inflammasome activation (Taxman et al., 2006; Park et al., 2014; Yamaguchi et al., 2017). Park et al. (2004) found that *P. gingivalis* induced IL-1β through activation of Caspase-1 and NLRP3 in THP-1 cells, mediated by ATP release, P2X7 receptors, and lysosomal destruction. This inconsistency in experimental data characterizing the effects of *P. gingivalis* on NLRP3 and IL-1β expression may be related to difference in cell lines or experimental designs and procedures.

Growing evidence supports the role of a Caspase-11 (a functional ortholog of Caspase-4/5 in humans)-dependent non-canonical pathway in IL-1β secretion (Rathinam et al., 2012; Russo et al., 2018). For example, Caspase-11 has been reported to be essential for the inflammatory response to Gram-negative bacteria such as *Escherichia coli* and *Salmonella* in human (Kayagaki et al., 2011; Broz et al., 2012), potentially mediated *via* NLRP3-ASC-dependent Caspase-1 activation and IL-1β maturation. In this role as a positive regulator of the NLRP3 signal cascade, Caspase-11 directly binds to the lipid A portion of intracellular lipopolysaccharides (LPS) *via* its CARD domain, mediating LPS recognition and oligomerization (Shi et al., 2014). Furthermore, it has also been demonstrated that non-canonical Caspase-11 activation contributes to macrophage death in the absence of CASP1 during *Salmonella typhimurinm* infection, thus affecting a more direct and robust destruction (Broz et al., 2012). Therefore, since *P. gingivalis* is also an intracellular, Gram-negative microorganism, the specific role of Caspase-11 in *P. gingivalis*-mediated activation of NLRP3 inflammasomes warrants study.

*Streptococcus mitis* (*S. mitis*) is among the most abundant of the commensal bacterial taxa in healthy oral microbiomes (Aas et al., 2005), though its interaction with the host innate immune system is still poorly characterized. Although only a few toxins and virulence factors have been identified in *S. mitis*, recent study has shown its ability to enter human gingival fibroblasts (Di Nisio et al., 2015) and human macrophages (Di Giulio et al., 2018). Our preliminary research confirmed its entry into THP-1 cells (Supplementary Figure 1). Moreover, *S. mitis* has been shown to exert a strong immunomodulatory effect on human cells. This species has been reported to induce the expression of human β-defensin 2 and modulate the expression of IL-8 in gingival epithelial cells (Eberhard et al., 2009). We thus sought to test if NLRP3 inflammasomes were activated upon *S. mitis* invasion.

In order to better acknowledge the expression of each component of NLRPs inflammasome as well as IL-1β in the process of periodontitis, clinical samples from patients with periodontitis were analyzed in this study. In addition, human THP-1 cells were challenged with live *P. gingivalis* or *S. mitis* to further understand the specific role of NLRP3 inflammasome activation on dependence of Caspase-4 or Caspase-1 with the interaction of different periodontal microbes and to validate our hypothesis that the NLRP3 inflammasome activation pathway serves as a crucial step in recognition and discrimination between commensal (*S. mitis*) and pathogenic (*P. gingivalis*) bacteria. Furthermore, we propose that NLRP3 inflammasome activation is necessary for transduction of signals to the immune system in order to maintain homeostasis in periodontal tissues. This study lays a foundation for further exploration of a potential role of *S. mitis* in modulating the NLRP3 inflammasome response, and provides experimental data characterizing the genetic mechanisms underlying *P. gingivalis* mediation of inflammasome activation.

## Materials and Methods

### Gingiva Tissue Preparation

Gingival tissue specimens (*n* = 10) were obtained from healthy subjects (*n* = 5) and patients with severe chronic periodontitis (*n* = 5). The gingival samples from the healthy group were obtained during the extraction of premolars for orthodontic indications or erupted third molars. For the periodontitis groups, the gingival samples were collected during the extraction of hopeless teeth with severe chronic periodontitis. The inclusion and exclusion criteria are listed in Supplementary Table 1 (Experts Committee of Chinese Stomatological Association division of Periodontology, 2017). The samples were obtained and immediately fixed in 10% formalin for immunohistochemistry assays. This study was approved by the Research Ethics Committee of the Affiliated Stomatology Hospital of Zhejiang University School of Medicine (Ethics Approval No. 2019-74R) and carried out in accordance with the approved guidelines with all subjects gave written informed consent in accordance with the Declaration of Helsinki.

### Bacterial Culture

*P. gingivalis* (ATCC 33277) was grown on brain heart infusion (BHI) agar supplemented with 5% defibrinated sheep blood containing 5 mg/ml hemin and 0.5 mg/ml vitamin K (3-phytyl-menadione) under anaerobic conditions at 37°C. *S. mitis* (ATCC 49456) was grown on Tryptic Soy Broth (TSB) anaerobically. An optical density of 0.5 at 630 nm was determined to correlate to 5 × 10^8^ CFU/ml. The bacteria were washed and re-suspended in RPMI 1640 medium prior to the treatment of human THP-1 cells.

### THP-1 Cell Culture

Human acute monocytic leukemia (THP-1) cells (ATCC TIB-202) were grown in RPMI 1640 medium (Gibco, Thermo Fisher Scientific) supplemented with 10% fetal bovine serum (FBS; EveryGreen, TianhangBio, Hangzhou, China). Around 2 × 10^6^ cells in each well were differentiated into macrophage-like cells by treatment with 100 nm phorbol-12-myristate-13-acetate (PMA) (Sigma, St Louis, MO, USA) overnight. The cells were infected with live *P. gingivalis* or *S. mitis* at a MOI of 1:0.5, 1:5, or 1:50 for 2, 6, or 24 h, which was selected due to good cell viability (Supplementary Figure 2). According to the results of dose-dependent experiments, MOI = 50 was selected for application during time course assays with sampling at 2, 6, and 24 h or 1, 2, 6, and 24 h. In some experiments, the cells were co-stimulated with 5 mM ATP (Sigma) during bacterial infection. To inhibit NLRP3 inflammasome, cells were pretreated with MCC950 (S7809, Selleck, CN) for 1 h with indicated doses before stimulation.

### Cytokine Analysis

Mature IL-1β and active Caspase-1 levels were measured by ELISA (R&D System Inc., Minneapolis, MN, USA) using a microplate reader (Bio-Rad, CA, USA) following the manufacturer’s instruction and protocols. Culture supernatants were collected and stored at −80°C.

### RNA Interference Assay

Human small interfering RNAs (siRNAs) for NLRP3, CASP1, CASP4, and ASC were obtained from GenePharma (Shanghai, China). The efficiency of each siRNA was tested, and the optimal sequences were selected (Supplementary Figure 3, Supplementary Table 2). The cells were transfected with siRNA oligonucleotides (300 pmol) for 24 or 48 h using Lipofectamine 2000 transfection reagent (Invitrogen, Carlsbad, CA, USA) following the instructions of the transfection kit.

### Reverse Transcription and Quantitative Real-Time Polymerase Chain Reaction (qRT-PCR)

Total RNA was extracted from THP-1 cells with Trizol reagent (Invitrogen) according to the manufacturer’s instructions. RNA concentrations and purity were assessed using a NanoDrop ND-1000 spectrophotometer (NanoDrop Technologies, Rockland, DE, USA) and reverse transcribed into cDNA with a PrimeScript RT Master Mix reverse transcription kit (Takara, Japan). qRT-PCR was undertaken with an ABI 7500 real-time PCR system (Applied Biosystems, Foster City, CA, USA) using SYBR master mix (Takara) and an amplification program consisting of 30 s pre-incubation at 95°C, 40 cycles of 5 s at 95°C, and 34 s at 60°C, with GAPDH as an internal control. The results were analyzed by the comparative Ct method (2^-ΔΔCt^ formula). Primers were synthesized and purchased from Invitrogen (Supplementary Table 3).

### Western Blot Analysis

Cells were washed in ice-cold phosphate-buffered saline (PBS) and lysed in RIPA buffer (Cell Signaling Technology, Beverly, MA, USA). Each sample (30 μg) was subjected to separation by 12% SDS-PAGE at 120 V for 90 min, followed by transfer of the SDS-PAGE bands to polyvinylidene fluoride (PVDF) membranes (R&D system Inc., Minneapolis, MN, USA) under transfer conditions of 250 mA for 130 min. The membranes were probed with the following antibodies: anti-NLRP3 (#13158, Cell Signaling Technology); anti-IL-1β (#16806-1-AP, Proteintech, Chicago, IL, USA); anti-ASC (#10500-1-AP, Proteintech); anti-pro-Caspase-1 (#ab179515, Abcam, Cambridge, UK); and anti-Caspase-4 (#11856-1-AP, Proteintech). The secondary antibodies (HuaBio, Hangzhou, China) were used according to the isotype of primary antibodies. The bands were visualized using SuperSignal™ West Pico Chemiluminescent Substrate (34087, Thermo Fisher Scientific). Protein levels were determined by densitometric quantification *via* NIH Image J software normalized to β-actin as a loading control.

### Immunohistochemistry

All samples were dehydrated in graded alcohol after 24 h of fixation in 10% formalin, cleared in xylene, and embedded in paraffin. Sections were cut into 5-mm thickness, and the slides were subjected to immunohistochemistry. For antigen retrieval, the slides were immersed in EDTA (pH 8.0) and boiled for 20 min. After being washed repeatedly in PBS, the slides were incubated with the following primary antibodies: anti-NLRP1 (diluted 1:200; #12256-1-AP; Proteintech), anti-NLRP2 (diluted 1:200; #15182-1-AP; Proteintech), anti-NLRP3 (diluted 1:50; #19771-1-AP; Proteintech), anti-NLRP6 (diluted 1:50; #37752; Signalway Antibody; US), anti-NLRP12 (diluted 1:50; #37750; Signalway Antibody), anti-absent in melanoma 2 (AIM2) (diluted 1:50; #ab93015; Abcam), anti-IL-1β (diluted 1:200; #16806-1-AP; Proteintech), anti-ASC (diluted 1:200; #10500-1-AP; Proteintech), or anti-Caspase-1 (diluted 1:50; #ab62698; Abcam). After incubation in a humidified tray at 4°C overnight, the tissue was further treated with secondary antibody for 30 min at room temperature. The slides were then washed in PBS and mounted permanently. For the negative control, only the secondary antibody was used. Non-specific binding was not seen (Supplementary Figure 4). The layers of gingival epithelium and lamina propria were observed separately and analyzed to quantify of the expression of targeted protein. Within each area, five individual, non-overlapping areas at magnification of 400× were randomly selected by the same examiner, who viewed them under a video camera that was connected to a light microscope. The quantitative analysis of targeted protein was performed by assessing the average positive stained area percentage measured using the NIH Image J software.

### Statistical Analysis

At the analysis stage, the Student’s *t*-test method was used for the comparisons of each marker in immunohistochemistry between the periodontitis group and the healthy control. *In vitro* study, statistical analysis were conducted using one-way ANOVA (Dunnett-*t* test or Bonferroni and least significant difference methods) as indicated, if both the normality test and the test of homogeneity of variances were justified. Otherwise, a non-parametric test was used. All analyses mentioned above were conducted with IBM SPSS 23.0. Values of *p* < 0.05 were considered significant. Data shown are representative of experiments done at least three times. Data are presented as mean ± standard deviation (SD).

## Results

### IL-1β and NLRP3 Inflammasome Were Highly Expressed in Gingival Tissues With Severe Chronic Periodontitis

**Figure 1:**
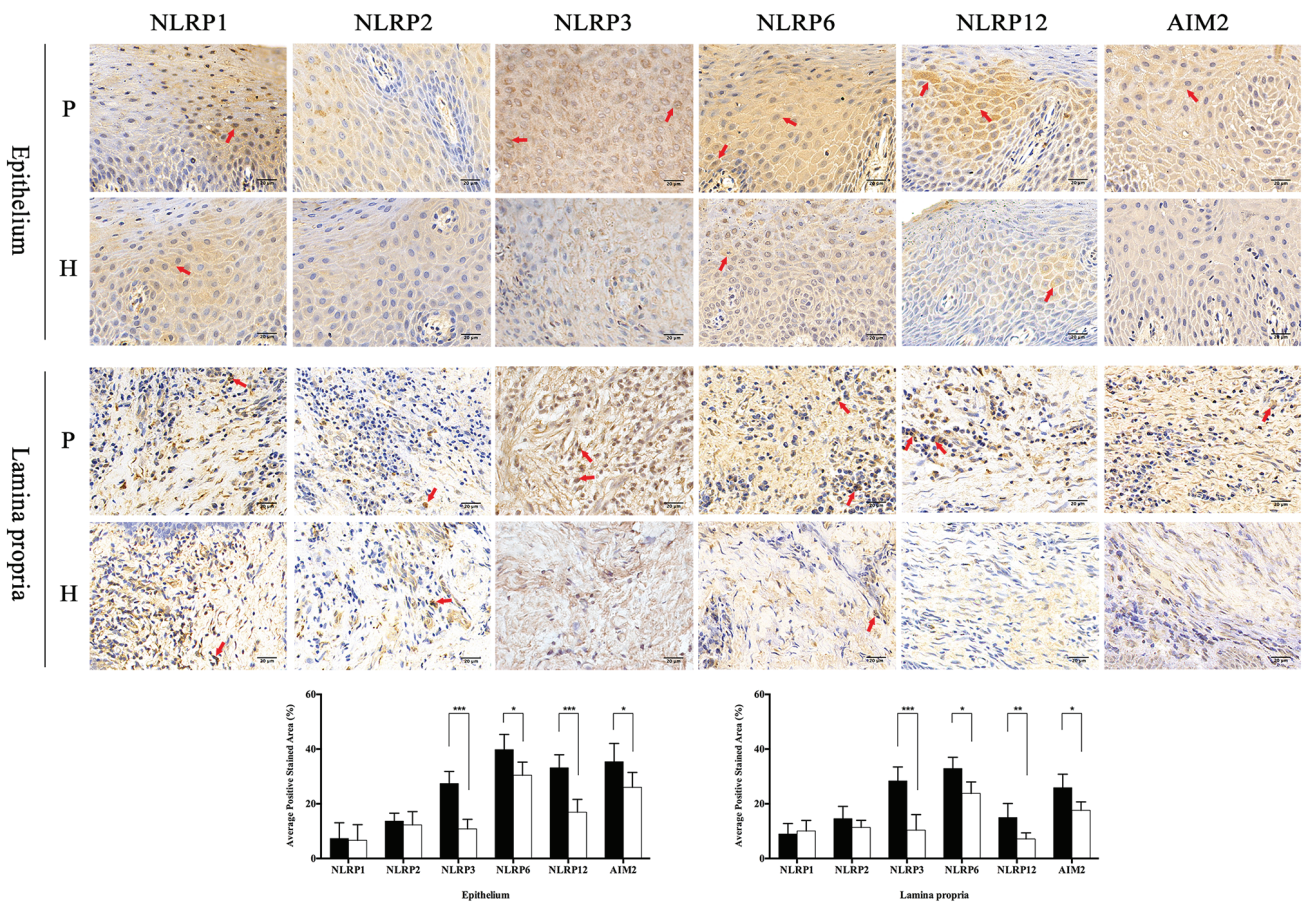
The distribution of NLRP1, NLRP2, NLRP3, NLRP6, NLRP12, and absent in melanoma 2 (AIM2) in the gingival epithelium and lamina propria of healthy and periodontitis patients. Full-thickness gingival tissue specimens including both the epithelium and connective tissue were obtained from healthy subjects (*n* = 5) and patients with severe chronic periodontitis (*n* = 5). Samples were fixed in 10% formalin for immunohistochemistry assays to detect the expression of NLRP1, NLRP2, NLRP3, NLRP6, NLRP12, and AIM2 (P: periodontitis specimens, *n* = 5; H: healthy gingiva, *n* = 5; arrows: positive stain; and magnification: 400×). ^*^p < 0.05, ^**^p < 0.01, and ^***^p < 0.001.

**Figure 2:**
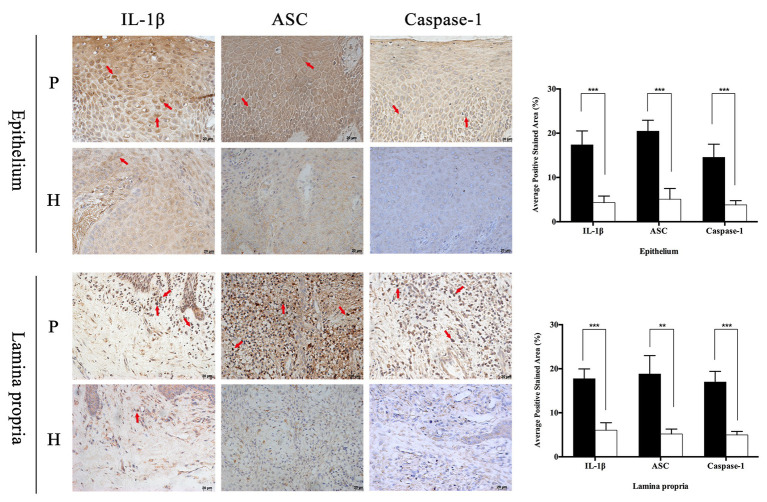
Periodontitis induced higher expression of interleukin-1β (IL-1β), apoptosis-related point-like protein (ASC), and Caspase-1 in gingival tissues. Full-thickness gingival tissue specimens including both the epithelium and connective tissue were obtained from healthy subjects (*n* = 5) and patients with severe chronic periodontitis (*n* = 5). Samples were fixed in 10% formalin for immunohistochemistry assays to detect the expression of IL-1β, ASC, and Caspase-1 (P: periodontitis specimens, *n* = 5; H: healthy gingiva, *n* = 5; arrows: positive stain; and magnification: 400×). ^*^p < 0.05, ^**^p < 0.01, and ^***^p < 0.001.

In order to observe the different distribution of NLRP family intuitively, immunohistochemistry was applied to analyze the expression characteristics of NLRP1, NLRP2, NLRP3, NLRP6, and NLRP12 in full-thickness gingival tissue samples obtained from severe periodontitis patients and healthy people. In addition, another inflammasome AIM2 was also detected. As shown in Figure 1, the distribution of these proteins varied greatly between periodontitis and healthy group. It appeared that the expression of NLRP1 and NLRP2 were both slight and had no big difference in two groups. However, NLRP3, NLRP6, NLRP12, and AIM2 were expressed much higher in gingival tissues with periodontitis than in healthy group, especially for NLRP3 and NLRP12. Moreover, NLRP12 was mostly distributed in the epithelium, while cells stained for NLRP3 were observed throughout the epithelium and lamina propria in periodontitis tissues. Similarly, we found the staining of IL-1β, ASC, and Caspase-1 was also significantly stronger in periodontitis group (Figure 2). The staining for NLRP3, ASC, Caspase-1, and IL-1β was widely distributed throughout the prickle cell layer in epithelium. And within the lamina propria of periodontitis tissue, the recruited inflammatory cells like plasma cells and macrophages, and gingival fibroblasts were positive stained. Conversely, cells in healthy gingiva presented a negative or very slight staining of IL-1β, NLRP3, ASC, and Caspase-1 (Figure 2). The results showed that these NLRP3 related proteins were highly expressed in periodontitis and that their roles are worth further investigation.

### *P. gingivalis* Promoted the Synthesis and Secretion of IL-1β More Robustly Than *S. mitis*

**Figure 3:**
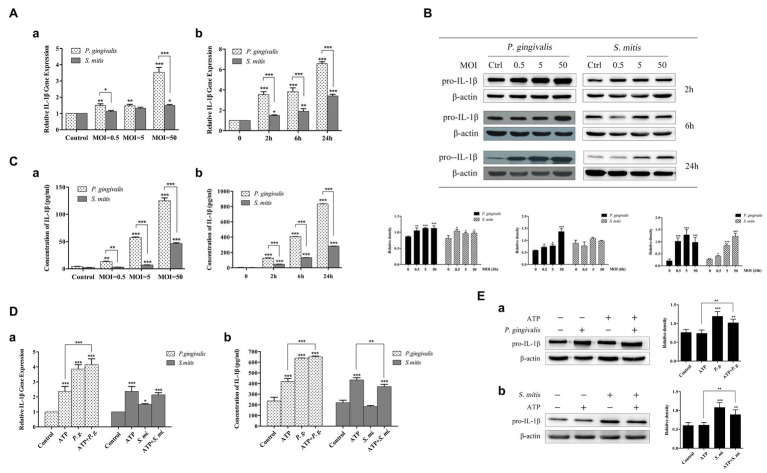
Treatment with *Porphyromonas gingivalis* more effectively stimulated production of IL-1β messenger RNA (mRNA), pro-IL-1β, and IL-1β protein than *Streptococcus mitis*. Adenosine 5'-triphosphate (ATP) in conjunction with *P. gingivalis*, but not *S. mitis*, exhibited an enhanced signaling on the induction of IL-1β expression. **(A–C)** Dose-dependent (MOI = 0.5, 5, and 50) and time course (2, 6, and 24 h) assay of phorbol-12-myristate-13-acetate (PMA)-primed THP-1 cells infected with *P. gingivalis* or *S. mitis*. IL-1β mRNA expression was measured by real-time qPCR **(Aa,b)**, intracellular pro-IL-1β was detected by immunoblotting **(B)**, and mature IL-1β secreted into supernatant was assayed by ELISA **(Ca,b)**. **(D–E)** PMA-primed THP-1 cells were infected with *P. gingivalis* or *S. mitis* (MOI = 50) with or without ATP for 2 h. IL-1β mRNA expression was measured by real-time PCR **(Da)**, mature IL-1β secreted into supernatant was assayed by ELISA **(Db)**, and intracellular pro-IL-1β was detected by immunoblotting **(Ea,b)**. Data of real-time qPCR and ELISA represent means ± SD of at least three independent experiments and results of immunoblot analysis were representative of at least three experiments. ^*^p < 0.05, ^**^p < 0.01, and ^***^p < 0.001.

In order to better understand the relationship between IL-1β expression and infection by *P. gingivalis* and *S. mitis*, we conducted a dose-dependent (MOI = 0.5, 5, and 50) time course (2, 6, and 24 h) to measure expression of IL-1β in human THP-1 cells in response to exposure to *P. gingivalis* and *S. mitis* using qRT-PCR, western blot, and ELISA analysis. We found that *P. gingivalis* induced pro-IL-1β mRNA and protein levels gradually, over time, and at all doses (Figures 3A,B). However, *S. mitis* only increased pro-IL-1β gene expression at MOI = 50 with time, and to significantly lower levels than *P. gingivalis* (Figure 3A). *S. mitis* also increased pro-IL-1β protein at 2 and 24 h at all doses (Figure 3B). In contrast, *P. gingivalis* promoted the release of mature IL-1β in a dose‐ and time-dependent manner, with the highest peak at the MOI of 50 at 24 h (Figure 3C). Although there was a similar increase in *S. mitis*-induced IL-1β secretion, this activity was much lower than that of *P. gingivalis* (Figure 3C). The above results suggest that *P. gingivalis* may promote the synthesis and secretion of IL-1β much more efficiently than *S. mitis*.

Since extracellular ATP has also been implicated in the activation of NLRP3 inflammasomes during *P. gingivalis* infection, we also investigated whether exposure to exogenous ATP affected the expression of IL-1β induced by these two strains (MOI = 50). Upon treatment with ATP, a significant increase was observed in IL-1β gene expression (Figure 3Da) as well as its secretion (Figure 3Db), though pro-IL-1β remained unchanged (Figure 3E). Interestingly, in the presence of *P. gingivalis*, both IL-1β gene expression and supernatant secretion were significantly elevated compared with treatment by ATP alone. However, *S. mitis* showed a slight inhibitory effect on ATP-induced IL-1β gene expression and protein accumulation (Figure 3D). Both *P. gingivalis* and *S. mitis* up-regulated pro-IL-1β protein expression with or without the addition of ATP (Figure 3E).

### NLRP3 and Caspase-1 Expression Mediated by *P. gingivalis* Was Enhanced After ATP Addition While *S. mitis* Alleviated the Effect Caused by ATP

**Figure 4:**
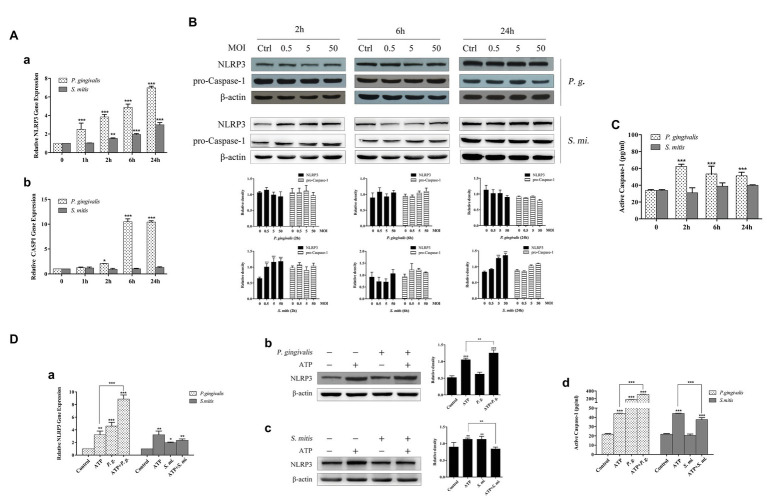
Caspase-1 expression was activated more efficiently by *P. gingivalis* than by *S. mitis*. Treatment with ATP led to significant upregulation of transcription and protein expression for NLRP3 and active Caspase-1. ATP combined with *P. gingivalis* induced an even higher expression of NLRP3 and Caspase-1 while *S. mitis* did not. **(A–C)** PMA-primed THP-1 cells were infected with *P. gingivalis* or *S. mitis* (MOI = 50) for 1, 2, 6, and 24 h. NLRP3 and Caspase-1 mRNA expression was measured by real-time qPCR **(Aa,b)**, intracellular protein was detected by immunoblotting **(B)**, and activated Caspase-1 secreted into supernatant was assayed by ELISA **(C)**. **(D)** PMA-primed THP-1 cells were infected by *P. gingivalis* or *S. mitis* (MOI = 50) with or without ATP for 2 h. NLRP3 mRNA expression was measured by real-time qPCR **(Da)**, NLRP3 protein was detected by immunoblotting **(Db,c)** and activated Caspase-1 secreted into supernatant was assayed by ELISA **(Dd)**. Real-time qPCR data and ELISA represent means ± SD of at least three independent experiments and immunoblot analysis results were representative of at least three experiments. ^*^p < 0.05, ^**^p < 0.01, and ^***^p < 0.001.

To examine how development of NLRP3 inflammasomes was affected by infection with *P. gingivalis* or *S. mitis*, we measured the expression of NLRP3, CASP1, and ASC (Supplementary Figure 5) at 1, 2, 6, and 24 h (MOI = 50) time points. The qRT-PCR assays showed that the expression of NLRP3 was up-regulated in a time-dependent manner during exposure to *P. gingivalis*, while mRNA transcription increased within 2 h of exposure to *S. mitis* (Figure 4Aa) in THP-1 cells. In contrast, CASP1 mRNA expression sharply spiked at 6 and 24 h during exposure to *P. gingivalis* but remained at baseline expression in the presence of *S. mitis* (Figure 4Ab). In terms of protein accumulation, NLRP3 significantly increased following inoculation with *S. mitis* at 2 h (MOI = 0.5, 5, and 50) and at 24 h (MOI = 5 and 50). However, inoculation with *P. gingivalis* did not affect NLRP3 protein levels at any dose or time point (Figure 4B), but ASC levels significantly dropped at 2 h (MOI = 5 and 50) and increased at 6 h (MOI = 0.5, 5, and 50), then significantly dropped again by 24 h (MOI = 0.5, 5, and 50; Supplementary Figure 5B). While pro-caspase-1 was not significantly different from unexposed cells over time or among MOIs for either *P. gingivalis* or *S. mitis* (Figure 4B), active Caspase-1 was significantly elevated by *P. gingivalis*, but not by *S. mitis* (Figure 4C).

Given that ATP is also reported to act as a signal in the NLRP3-mediated inflammasome pathway, we evaluated whether treatment with ATP affected the expression and accumulation of NLRP3 in the presence of these two bacterial strains (MOI = 50). It showed that NLRP3 mRNA and protein were both elevated during treatment with ATP, though to a much greater extent in THP-1 cells treated with *P. gingivalis*, and reached an even higher level after ATP addition in stimulating NLRP3 expression (Figure 4Da,b). This effect was similar to our previous observations of IL-1β expression, and as with IL-1β, NLRP3 expression was also inhibited by co-treatment with *S. mitis* compared to its expression during treatment with either ATP or *S. mitis* alone (Figure 4Dc). In light of these data, we examined the concentration of active Caspase-1 and found that it was increased by exposure to ATP and by an order of magnitude or greater by treatment with *P. gingivalis*, alone or in conjunction with ATP, while treatment with *S. mitis* had no effect at all (Figure 4Dd).

### Knockdown of NLRP3 Leads to Differential Responses to *P. gingivalis* and *S. mitis* in the Induction of IL-1β

**Figure 5:**
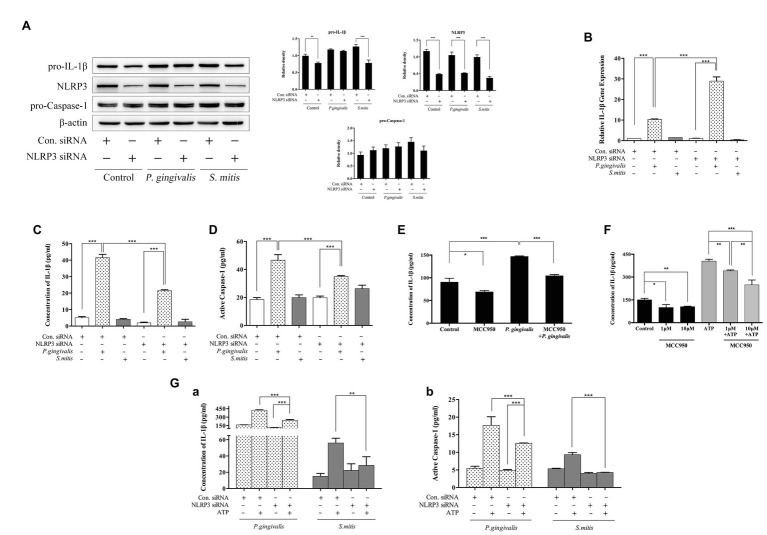
NLRP3 was necessary for *P. gingivalis*-, but not *S. mitis*-induced IL-1β production in THP-1 cells. **(A–D)** PMA-primed THP-1 cells were transfected with NLRP3 small interfering RNA (siRNA) for 24 (gene detection) or 48 h (protein detection). siRNA-transfected cells were infected with *P. gingivalis* or *S. mitis* (MOI = 50) for 2 h. Pro-IL-1β, NLRP3, pro-CASP1, and β-actin in cell lysates were detected by immunoblotting **(A)**. IL-1β mRNA expression was measured by real-time qPCR **(B)**. Cell culture supernatant was collected and assayed for IL-1β **(C)** and active Caspase-1 **(D)** secretion by ELISA. **(E)** PMA-primed THP-1 cells were pre-treated with 10 μm MCC950 and then infected by *P. gingivalis* of MOI 50 for 2 h. **(F)** PMA-primed THP-1 cells were pre-treated with 1 and 10 μm MCC950 followed by ATP stimulation for 2 h. **(G)** PMA-primed THP-1 cells were transfected with NLRP3 siRNA for 48 h and then treated by *P. gingivalis* or *S. mitis* (MOI = 50) with or without ATP for 2 h. Both IL-1β **(Ga)** and active Caspase-1 secretion **(Gb)** were detected by ELISA. Real-time qPCR data and ELISA represent means ± SD of at least three independent experiments and immunoblot analysis results were representative of at least three experiments. ^*^p < 0.05, ^**^p < 0.01, and ^***^p < 0.001.

To characterize the specific role of NLRP3 in the induction of IL-1β expression by *P. gingivalis* or *S. mitis*, we selected an NLRP3-targeting siRNA (Supplementary Figure 3A) to knock down NLRP3 expression in PMA-primed THP-1 cells before bacterial challenge. With the clear decrease in NLRP3 expression (Figure 5A), IL-1β mRNA levels were significantly increased (~3-fold) by treatment with *P. gingivalis*, but not by treatment with *S. mitis*, in NLRP3 knockdown cells compared to control cells (Figure 5B). Intracellular pro-IL-1β expression was comparably decreased in untreated and *S. mitis*-treated knockdown cells, while both control and NLRP3 knockdown cells showed no difference in pro-IL-1β content during *P. gingivalis* infection (Figure 5A). Moreover, IL-1β secretion level (Figure 5C) and active Caspase-1 content (Figure 5D), though increased by treatment with *P. gingivalis* compared to untreated cells, were significantly lower in NLRP3-silenced cells than in unsilenced cells when both were treated with *P. gingivalis*. This was also consistent with the results of using NLRP3 inflammasome inhibitor MCC950 (Figures 5E,F). This finding indicates that NLRP3 is necessary for activation of Caspase-1 and release of IL-1β in response to *P. gingivalis*. In contrast, *S. mitis* did not elicit a significant change in IL-1β and Caspase-1 content in either knockdown or control cells (Figures 5C,D). These findings strongly suggest that since NLRP3 expression is induced by *P. gingivalis* and *S. mitis*, and that knockdown of NLRP3 leads to a less downstream activation of IL-1β and active Caspase-1 expression by *P. gingivalis*, but not activation by *S. mitis*, NLRP3 is likely essential for the recognition pathway that discriminates between *P. gingivalis* and *S. mitis*.

In addition to microbial components, danger associated molecular patterns (DAMPs) such as extracellular ATP can also activate NLRP3 inflammasomes. In order to determine if the response to DAMPs was also mediated by NLRP3, we measured differences in the protein levels of IL-1β and active Caspase-1 in the NLRP3 knockdown THP-1 cells with and without exposure to ATP. Active Caspase-1 and mature IL-1β were both increased in unsilenced control cells after co-stimulation with ATP and *P. gingivalis*, and silencing of NLRP3 slightly diminished this response. Co-treatment with ATP and *S. mitis* increased IL-1β and active Caspase-1 to a less extent than that *P. gingivalis* in control cells, but the response to ATP was indistinguishable from untreated controls in NLRP3-silenced cells during exposure to *S. mitis* (Figure 5G). Generally, these results confirm that NLRP3 is necessary for Caspase-1 activation and IL-1β secretion in response to extracellular ATP. Moreover, *P. gingivalis* more efficiently induced the accumulation of Caspase-1 and IL-1β compared to *S. mitis* in the presence of ATP, even under weak expression of NLRP3.

### Knockdown of CASP1 Leads to Differential Induction of IL-1β Production in Response to *P. gingivalis* and *S. mitis*

**Figure 6:**
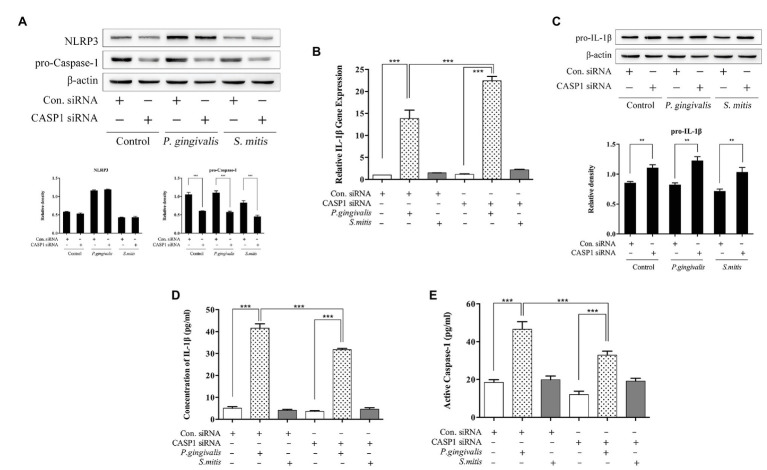
Caspase-1 plays a substantial role during *P. gingivalis*-, but not *S. mitis*-induced IL-1β production in THP-1 cells. **(A–E)** PMA-primed THP-1 cells were transfected with CASP1 siRNA for 24 (gene detection) or 48 h (protein detection). siRNA-transfected cells were infected with *P. gingivalis* or *S. mitis* (MOI = 50) for 2 h. Pro-IL-1β, NLRP3, pro-CASP1, and β-actin in cell lysates were detected by immunoblotting **(A,C)**. Real-time PCR measurements of IL-1β mRNA expression after treatment **(B)**. Cell culture supernatant was collected and assayed for IL-1β and active Caspase-1 secretion by ELISA **(D,E)**. Data from real-time qPCR and ELISA represent means ± SD of at least three independent experiments and immunoblot analysis results were representative of at least three experiments. ^*^p < 0.05, ^**^p < 0.01, and ^***^p < 0.001.

In order to understand the relationship between Caspase-1 and IL-1β expression induced by *P. gingivalis* or *S. mitis*, we used a targeted siRNA to successfully knockdown pro-caspase-1 expression in THP-1 cells before bacterial challenge (Supplementary Figure 3B and Figure 6A). Caspase-1 silencing had no effect on NLRP3 protein expression (Figure 6A). While, it increased IL-1β mRNA expression by *P. gingivalis* stimulation only, similar to the pattern observed under NLRP3 silencing (Figure 6B). However, pro-IL-1β protein levels uniformly increased in both treatment and control groups after CASP1 knockdown (Figure 6C). Moreover, *P. gingivalis* treatment of CASP1-silenced cells led to significantly lower IL-1β concentration than did the same treatment of unsilenced cells (Figure 6D), as was also the case with Caspase-1 activation (Figure 6E); whereas treatment with *S. mitis* failed to increase the levels of either protein in both silenced and control cells (Figures 6D,E). Due to silencing of CASP1, the further increase in pro-IL-1β induction during challenge with *S. mitis*, indicates a role for Caspase-1 in the expression of this gene, though not in the induction of mature IL-1β mRNA and protein. In contrast, these data showed an elevation of IL-1β transcription and reduced induction of IL-1β protein in CASP1-silenced cells, indicating that Caspase-1 is necessary for synthesis of IL-1β in response to challenge with *P. gingivalis*.

### Caspase-4 Was Engaged in Regulating IL-1β Production Induced by *P. gingivalis* but Not *S. mitis*

**Figure 7:**
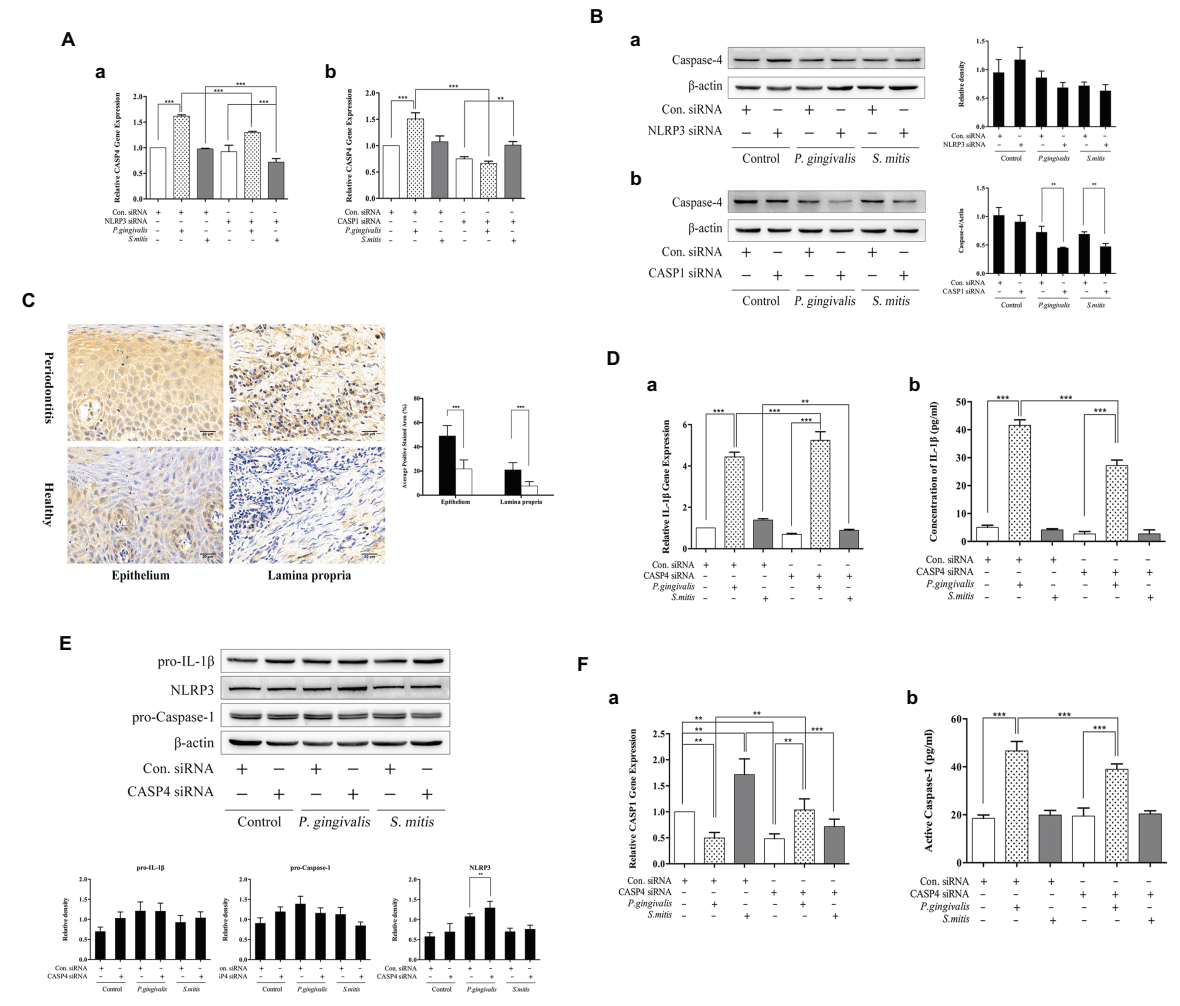
Caspase-4 (CASP4) played a substantial role during *P. gingivalis*, not *S. mitis*-induced IL-1β production in THP-1 cells. **(A,B)** PMA-primed THP-1 cells were transfected with NLRP3 or CASP1 siRNA for 24 (gene detection) or 48 h (protein detection). siRNA-transfected cells were treated with *P. gingivalis* or *S. mitis* (MOI = 50) for 2 h. Real-time qPCR measurements of Caspase-4 mRNA expression after infection **(Aa,b)**. Caspase-4 in cell lysates were detected by immunoblotting **(Ba,b)**. **(C)** Full-thickness gingival tissue specimens including both the epithelium and connective tissue were obtained from healthy subjects (*n* = 5) and patients with severe chronic periodontitis (*n* = 5). Samples were fixed in 10% formalin for immunohistochemistry assays to detect the expression of Caspase-4. **(D–F)** PMA-primed THP-1 cells were transfected with CASP4 siRNA for 24 (gene detection) or 48 h (protein detection). siRNA-transfected cells were treated with *P. gingivalis* or *S. mitis* (MOI = 50) for 2 h. Real-time qPCR measurements of IL-1β and Caspase-1 mRNA expression after infection **(Da,Fa)**. Pro-IL-1β, NLRP3, pro-CASP1, and β-actin in cell lysates were detected by immunoblotting **(E)**. Cell culture supernatant was collected and assayed for IL-1β and active Caspase-1 secretion by ELISA **(Db,Fb)**. Data from real-time qPCR and ELISA represent means ± SD of at least three independent experiments and immunoblot analysis results were representative of at least three experiments. ^*^p < 0.05, ^**^p < 0.01, and ^***^p < 0.001.

Since it remains undetermined how murine Caspase-11 or human Caspase-4 function in the activation of NLRP3 inflammasomes in response to pathogenic or commensal periodontal bacteria, we examined the levels of Caspase-4 mRNA and protein in human THP-1 cells during siRNA silencing of either NLRP3 or CASP1. We found that Caspase-4 was transcriptionally up-regulated only upon *P. gingivalis* infection but down-regulated in both NLRP3‐ and Caspase-1-silenced cells (Figure 7A). Treatment with *S. mitis* did not increase Caspase-4 mRNA expression in unsilenced cells, though silencing of NLRP3 leads to a significant decrease in the Caspase-4 transcription in the presence of this commensal (Figure 7Aa). Moreover, Caspase-4 protein was significantly decreased in both *P. gingivalis* and *S. mitis* test groups by knockdown of Caspase-1 but not NLRP3 (Figure 7B). These results indicate that Caspase-4 may play a role in the inflammatory response to exposure to *P. gingivalis* and *S. mitis*. In order to observe the expression of Caspase-4 in periodontitis tissue more intuitively, we performed IHC for Caspase-4 staining in human gingival tissues. The staining for Caspase-4 was obviously stronger in both epithelium and lamina propria of periodontits group (Figure 7C), suggesting that Caspase-4 also participated in the mechanism of periodontitis.

We further examined the effects of silencing CASP4 on IL-1β transcription and protein expression to determine if CASP4 was necessary for its biosynthesis in the NLRP3 inflammasome pathway. We found that IL-1β mRNA levels increased during *P. gingivalis* infection but decreased in the presence of *S. mitis* in CASP4-silenced cells (Figure 7Da). While mature IL-1β secretion was significantly lower in *P. gingivalis*-treated CASP4-silenced and unsilenced control cells (Figure 7Db), although the intracellular pro-IL-1β showed no clear difference due to CASP4 silencing or bacterial exposure (Figure 7E). Moreover, we also checked for a change in the expression of NLRP3 inflammasome components under CASP4 silencing and found that NLRP3 protein was significantly increased during *P. gingivalis* infection compared to unsilenced controls (Figure 7E). In both control and *S. mitis* treatment groups, transcription of Caspase-1 decreased under CASP4 silencing, but increased during *P. gingivalis* infection (Figure 7Fa). However, active Caspase-1 levels were lower in CASP4-silenced cells compared to unsilenced cells when both were treated with *P. gingivalis* (Figure 7Fb), while *S. mitis* did not elicit a change in either silenced or control siRNA cells. These findings were consistent with the results of gene expression assays in both NLRP3 and CASP1 knockdown cells. We thus inferred that Caspase-4 is essential to *P. gingivalis*-induced expression of IL-1β, Caspase-1, and NLRP3. In contrast, these results show that Caspase-4 does not contribute to accumulation of IL-1β in response to *S. mitis* exposure, although it may participate in the transcriptional regulation of IL-1β and Caspase-1.

## Discussion

Our findings demonstrated that NLRP3 inflammasome and IL-1β were both highly expressed in human gingival tissues with severe chronic periodontitis. Furthermore, it showed that the secretion of IL-1β, induced by exposure to *P. gingivalis*, was regulated through a non-canonical Caspase-4-dependent pathway, in addition to the NLRP3/Caspase-1 canonical signaling pathway in human THP-1 cells. Meanwhile, the activities of two pathways could be potentially correlated through the interaction between Caspase-1 and Caspase-4 for enhanced danger signaling. Although our data showed that *S. mitis* can also increase IL-1β secretion, the mechanism for this response pathway is unrelated to the signaling pathway mediated by NLRP3/Caspase-1/Caspase-4. The above results indicate that NLRP3 inflammasome activation is a part of a crucial recognition pathway in order to maintain homeostasis in periodontal tissues and they may function by discriminating between pathogenic and commensal strains and initiating distinctly different immune responses during microbial invasion.

Recent studies have found higher expression of IL-1β as well as NLRP3 inflammasomes in gingival tissues of periodontitis compared to healthy tissues (Bostanci et al., 2009; Xue et al., 2015), which is consistent with our findings in gingival tissue samples. In the future study, extracting total protein from gingival tissue specimens or collecting human gingival cervical fluid to detect the expression of IL-1β quantitatively are highly recommended. *P. gingivalis* could promote NLRP3 and IL-1β but decreased ASC levels in MonoMac-6 cells (Bostanci et al., 2009). Our data further determined that *P. gingivalis* induced the up-regulation of IL-1β and NLRP3 transcription in PMA-primed THP-1 cells. While, we found that ASC protein expression induced by *P. gingivalis* was down-regulated at first (2 h) but elevated at 6 h, and finally decreased again. As ASC participates in cell death and bacterial clearance (Ohtsuka et al., 2004), its fluctuation may indicate that *P. gingivalis* was battling with the host to survive. Since pro-IL-1β was cleaved by active Caspase-1, whose precursor was recruited by NLRP3/ASC complex during the assembly of inflammasome, our results found active Caspase-1 and IL-1β protein were significantly elevated at all time points, indicating the elevated activation of NLRP3 inflammasome during exposure to *P. gingivalis*. Whereas, Taxman et al. (2012) found that *P. gingivalis* infected BMDMs could not process Caspase-1 activation at all time points (Taxman et al., 2012). This is possibly due to the different cells types we used. Many studies highlighted that a second signal ATP was needed for Caspase-1 activation and IL-1β maturation besides pathogenic stimulus (He et al., 2013; Ramos-Junior et al., 2015). However, THP-1 cells priming with PMA would cause constitutive release of endogenous ATP (Netea et al., 2009), which enabled NLRP3 inflammasome activation without extracellular ATP.

Recent studies also reported that active Caspase-1 was only stimulated at MOI of 10 and 100 but not 500 of *P. gingivalis* (Jun et al., 2017), as its virulence factor gingipains had contradictory functions in promoting Caspase-1 activation and the proteins released from Caspase-1-activated cells were rapidly degraded by gingipains (Jung et al., 2015). Moreover, prior research suggests that some variants of virulence factors found in *P. gingivalis*, such as LPS and fimbriae (Bostanci and Belibasakis, 2012) are recognized by toll-like receptors (TLRs) during infection, subsequently triggering the NF-κB pathway to up-regulate expression of pro-IL-1β and some NLRs, like NLRP3 (Bauernfeind et al., 2009). In addition, outer membrane vesicles (OMVs) shed from *P. gingivalis* could interact with monocytes and macrophages strongly including NF-κB activation and inflammasome activation (Cecil et al., 2017; Fleetwood et al., 2017). In this study, live *P. gingivalis* strains were used to better simulate the real periodontal environment and counteract the interaction of different virulence factors. Furthermore, our data showed decreased secretion of mature IL-1β, along with active Caspase-1, in both NLRP3‐ and CASP1-silenced THP-1 cells infected with *P. gingivalis,* though ASC remained unchanged in all groups. Park et al. (2014) also found decreased IL-1β and pyroptic cell death with NLRP3 knockdown during *P. gingivalis* infection (Park et al., 2014), so did in NLRP3 or ASC silencing cells stimulated by LPS (Netea et al., 2009). Taken together, these findings provide evidence for the activation of NLRP3 inflammasomes during *P. gingivalis* infection, accompanied by elevated processing of Caspase-1 and secretion of mature IL-1β, and ultimately, increased cytotoxicity. Although our gingival samples staining showed NLRP6, NLRP12, and AIM2 were also highly expressed in periodontitis tissue, the decrease of IL-1β secretion caused by ASC silencing was to a similar degree with NLRP3 silencing (Supplementary Figure 6D), indicating that NLRP3 inflammasomes activation was probably in a dominant position during *P. gingivalis* infection compared with other inflammasomes.

Since periodontal keystone pathogens like *P. gingivalis* can influence the host inflammatory response, the composition of the oral microbial community may be altered in the process, the result of which is dysbiosis of periodontium (Hajishengallis et al., 2011). Therefore, more complicated mechanisms must be considered to identify the salient features of *P. gingivalis* infection. Some studies have reported that a Caspase-4/Caspase-11-dependent, non-canonical pathway for NLRP3 inflammasome activation may be a critical step in the regulation of IL-1β secretion stimulated by the LPS of Gram-negative bacteria (Rathinam et al., 2012; Casson et al., 2015; Vigano et al., 2015). Furthermore, in contrast to the receptor/scaffold-mediated activation pathway (Rathinam et al., 2012), the direct binding of Caspase-11 to cytoplasmic LPS, independent of TLR4, can process its autoactivation (Hagar et al., 2013), suggesting an extreme sensitivity to LPS or bacteria challenge and a more rapid and direct immune response. Attenuation of the IL-1β responds to transfected LPS has been previously confirmed in HeLa and human THP-1 cells, as well as primary macrophages with siRNA knockdown and CRISPR/Cas9 deletion of Caspase-4 (Baker et al., 2015; Casson et al., 2015; Schmid-Burgk et al., 2015), indicating that Caspase-4 played a crucial role in LPS detection and IL-1β release. Interestingly, our findings revealed that *P. gingivalis*-induced IL-1β secretion was also regulated by Caspase-4 in THP-1 cells. Consistent with our study, Jun et al. (2017) also proved Caspase-4 activation by *P. gingivalis* and other bacteria including *Treponema denticola* and *Tannerella forsythia*. In addition, silencing of either CASP1 or CASP4 can lead to a concurrent or reciprocal decrease in the expression of the other, suggesting that their expression and activities are correlated. This interconnection is potentially explained by the finding that Caspase-4 binds to the p20 subunit of Caspase-1 for its activation in transfected COS-1 cells (Sollberger et al., 2012). Moreover, Caspase-4 cleaves Gasdermin D, the N-terminal subunits of which form pores in the cell membrane during pyroptosis (Ding et al., 2016; Gaidt and Hornung, 2016), consequently activating NLRP3 inflammasomes and Caspase-1 *via* K^+^ efflux (Kayagaki et al., 2015; Ruhl and Broz, 2015). Therefore, we are inclined to speculate that there are interactions between the canonical and non-canonical pathways for NLRP3 inflammasome activation, though whether or not Gasdermin D-mediated pyroptosis is also part of this mechanism requires further study.

Periodontal health is closely related to the structure of the commensal microbial community. The *mitis* group of *Steptococci* including, *Streptococcus gordonii*, *Streptococcus pneumoniae*, *S. mitis*, and *Streptococcus orali*, are part of an abundant and diverse oral microbiome (Nobbs et al., 2009). Although *S. mitis* has been characterized as one of the earliest commensal colonizers of oral mucosa and participates in biofilm formation, the relationship between periodontitis and commensals like *S. mitis* remains unclear. Engen et al. (2018) identified a set of genes encoding immunity-modulating molecules such as IL-1α, IL-1β, IL-10, and TNF, which are up-regulated in monocytes after *S. mitis* stimulation (Engen et al., 2018). Our study further determined that *S. mitis* also promotes the synthesis and secretion of IL-1β, but to a far lower extent than that induced by *P. gingivalis*. *S. mitis* may be recognized by TLR2 through lipoteichoic acids (Ellingsen et al., 2002), subsequently activating the NF-κB pathway to up-regulate transcription of pro-IL-1β and NLRP3. In addition, unlike the pathogenic *P. gingivalis*, the inflammatory response elicited by commensals like *S. mitis* is probably self-limiting (Engen et al., 2018), though the exact mechanism of which has not been resolved yet. However, based on our results wherein the silencing of NLRP3, CASP1, or CASP4 did not significantly affect the levels of mature IL-1β secretion during *S. mitis* infection, we conclude that NLRP3 inflammasome activation is not directly related to the mechanism by which *S. mitis* induces IL-1β release.

We found that using ATP as a co-stimulant with *P. gingivalis* induced higher levels of Caspase-1 as well as mature IL-1β than ATP alone, indicating an enhanced danger signaling. This is likely due to accelerated release of endogenous ATP (Lavieri et al., 2014) caused by ROS production (Asehnoune et al., 2004) after activation of TLR by *P. gingivalis*. Given that ATP can activate purogenic P2X7 ATP-gated ion channels (Kahlenberg and Dubyak, 2004), its release may contribute to K^+^ efflux and pannexin-1 recruitment, which then leads to the accumulation of extracellular agonists of NLRP3 in the cytoplasm, and subsequently, a direct activation of inflammasomes (Kanneganti et al., 2007).

Our study is also the first to report that treatment of THP-1 cells with *S. mitis* can lead to inhibition of ATP-induced activation of NLRP3 and Caspase-1. This finding suggests a potentially protective role for *S. mitis* against over-inflammation, which can subsequently alleviate local tissue damage. Another study also found that *S. mitis* was able to reduce the inflammatory response caused by *Pseudomonas aeruginosa* in acute lung infection, probably through modulation of TLR signaling (Song et al., 2017). However, *S. mitis* has also been reported as potentially pathogenic (Mitchell, 2011) due to its close taxonomic relationship to *S. pneumoniae* (Kilian et al., 2008) and its association with endocarditis or septicemia (Bochud et al., 1994). Our findings lead us to suspect that the exact role of *S. mitis* in modulating immune response may be closely related to specific external stimuli and the corresponding host immune functions that are activated in response. More in depth study is warranted to determine the specific mechanisms by which *S. mitis* interacts with its host, as well as its potential to mitigate *P. gingivalis*-induced inflammatory response.

In summary, our results demonstrated that NLRP3 inflammasomes may participate in a crucial process of discriminating between *P. gingivalis* and *S. mitis*. Despite *S. mitis*-induced IL-1β secretion was hardly related to the signaling pathway mediated by NLRP3/Caspase-1/Caspase-4, *P. gingivalis* could modulate NLRP3 inflammasome activation through both canonical Caspase-1-dependent and non-canonical Caspase-4 pathways, leading to a much more intense inflammatory response including Caspase-1 activation and IL-1β release during infection, which may crucially contribute to the dysregulated immuno-inflammatory response in periodontal pathogenesis.

## Data Availability Statement

The raw data supporting the conclusions of this article will be available on request to the corresponding author without undue reservation.

## Ethics Statement

The studies involving human participants were reviewed and approved by the Research Ethics Committee of the Affiliated Stomatology Hospital of Zhejiang University School of Medicine (Ethics Approval No. 2019-74R). The patients/participants provided their written informed consent to participate in this study.

## Author Contributions

Conceptualization, P-HD; methodology and validation, M-XY and N-NW; formal analysis, investigation, M-XY and N-NW; writing – original draft preparation, P-HD, M-XY and N-NW; writing – review and editing, P-HD and L-JJ; supervision, YD, XC and L-LC; project administration and funding acquisition, P-HD. All authors critically reviewed the manuscript before submission. All authors contributed to the article and approved the submitted version.

### Conflict of Interest

The authors declare that the research was conducted in the absence of any commercial or financial relationships that could be construed as a potential conflict of interest.

## Supplementary Material

SUPPLEMENTARY FIGURE S1 TEM micrographs of *S. mitis* entry into human THP-1 cells. Transmission electron microscope images of co-culture of *S. mitis* ATCC 49456 (MOI = 50) and THP-1 cells after 2 h. **(A)** *S. mitis* were found outside human THP-1 cells. **(B)** There were several chains of *S. mitis* between two cells without entry. **(C,D)** *S. mitis* cells were found inside THP-1 cells, localized near the cell membrane **(C)** or near the nucleus **(D)**. Arrow head: *S. mitis* chain outside cells; arrows: *S. mitis* chain inside cells. Magnification: 10,000× **(A,C)** and 11,500× **(B,D)**.

SUPPLEMENTARY FIGURE S2 Cell viability during *P. gingivalis* infection. Cell viability of human phorbol-12-myristate-13-acetate (PMA)-primed THP-1 cells was measured by CCK8 kit after *P. gingivalis* infection at 24 h for indicated MOIs. All doses of *P. gingivalis* exhibited little cell cytotoxicity after 24-h of infection.

SUPPLEMENTARY FIGURE S3 NLRP3, CASP1, CASP4, and ASC siRNA selection and evaluation of their efficiency. PMA-primed THP-1 cells (1 × 106) were transfected with three kinds of siRNA (three sequences per kind) for 24 h. Gene expression was measured by real-time qPCR. **(A)** NLRP3 siRNA-978 knocked down NLRP3 gene expression up to 57.8% and was selected for subsequent experiments. **(B)** CASP1 siRNA-853 knocked down CASP1 gene expression up to 69.6% and was selected for subsequent experiments. **(C)** CASP4 siRNA-801 knocked down CASP4 gene expression up to 94.6% and was selected for subsequent experiments. **(D)** ASC siRNA-651 knocked down ASC gene expression up to 96.6% and was selected for subsequent experiments. Data are representative of at least three experiments. ^*^*p* < 0.05, ^**^*p* < 0.01, and ^***^*p* < 0.001.

SUPPLEMENTARY FIGURE S4 Negative controls for NLRP1, NLRP2, NLRP3, NLRP6, NLRP12, absent in melanoma 2 (AIM2), IL-1β, ASC, Caspase-1, and Caspase-4 staining. Full-thickness gingival tissue specimens including both the epithelium and connective tissue were obtained from patients with severe chronic periodontitis. Sections were cut into 5-mm thickness and the slides were subjected to immunohistochemistry. After being washed repeatedly in phosphate-buffered saline (PBS), the slides were incubated with only the secondary antibody. Non-specific binding was not seen. Magnification: 100×.

SUPPLEMENTARY FIGURE S5 The expression of ASC after *P. gingivalis* or *S. mitis* treatment. PMA-primed THP-1 cells were treated with *P. gingivalis* or *S. mitis* (MOI = 50) for 1, 2, 6, and 24 h. ASC mRNA expression was measured by real-time qPCR **(A)**, ASC intracellular protein was detected by immunoblotting **(B)**. Both *P. gingivalis* and *S. mitis* induced a slight decreasing trend in ASC mRNA expression over the experimental time course **(A)**. In terms of protein accumulation **(B)**, ASC significantly increased following inoculation with *S. mitis* at 2 h (MOI = 0.5, 5, and 50) and at 24 h (MOI = 5 and 50). However, inoculation with *P. gingivalis*, ASC levels significantly dropped at 2 h (MOI = 5 and 50), increased at 6 h (MOI = 5 and 50), then significantly dropped again by 24 h (MOI = 0.5, 5, and 50) of exposure (Figure 2D). The trends for ASC accumulation thus showed an inverse relationship in comparisons between THP-1 treatment with *P. gingivalis* and *S. mitis*.

SUPPLEMENTARY FIGURE S6 The expression of ASC after *P. gingivalis* or *S. mitis* treatment in silenced cells. PMA-primed THP-1 cells were transfected with NLRP3, CASP1, CASP4, or ASC siRNA for 48 h. siRNA-transfected cells were treated with *P. gingivalis* or *S. mitis* (MOI = 50) for 2 h. ASC intracellular protein was detected by immunoblotting. NLRP3 or CASP4 silencing led to no significant change in ASC expression in all groups **(A,C)**. While in CASP1 silenced cells, ASC decreased during *S. mitis* treatment compared to unsilenced cells **(B)**. For ASC silencing cells, the secretion of mature IL-1β was examined by ELISA. It turned out that IL-1β was also decreased significantly when ASC silencing.
