# Supplementary material for: Porphyromonas gingivalis-Induced NLRP3 Inflammasome Activation and Its Downstream Interleukin-1β Release Depend on Caspase-4
Source: Front Microbiol. 2020 Aug 13;11:1881. doi: 10.3389/fmicb.2020.01881 (PMC7438778; doi:10.3389/fmicb.2020.01881)
Supplement: Supplementary file 1 [file Data_Sheet_1.DOCX]

**Supplementary Table 1. The inclusion and exclusion criteria for the selection of candidate patients.**

| Patients diagnosed with severe periodontitis must have two or more affected teeth with three characteristics listed below. If there are only 2 affected teeth left, they must be non-adjacent and in different quadrants. | | |
| --- | --- | --- |
| Inclusion criteria | **Severe Periodontitis** | 1. Probing depth (PD)> 6mm; 2. Attachment loss (AL) ≥ 5mm; 3. Alveolar bone absorption exceeds 1/2 of root length; 4. Loose teeth; 5. Apparent inflammation accompanied by periodontal abscess or not; 6. Degree II or III furcation lesions in posterior teeth. |
|  | **Healthy** | 1. No major systemic disease in physical health; 2. PD ≤ 3mm in periodontal examination, no loss of attachment, no alveolar bone loss; 3. Full mouth bleeding on probing (BOP) score does not exceed 15%; 4. no immunosuppressant in use; 5. no antibiotics within 3 months. |
| Exclusion criteria | ① systemic diseases, women who are pregnant or lactating;  ② have received periodontal treatment within six months;  ③ long-term use of immunosuppressants;  ④ antibiotics within 3 months. | |

**Supplementary Table 2. siRNA Sequences. NLRP3, CASP1, CASP4 and ASC siRNA selected to infect PMA-primed THP-1 cells for 24h or 48h before bacteria challenge.**

| Gene | Sense (5'-3') | Antisense (5'-3') |
| --- | --- | --- |
| NLRP3-homo-978 | CAACAGGAGAGACCUUUAUTT | AUAAAGGUCUCUCCUGUUGTT |
| CASP1-homo-853 | GCCCAAGUUUGAAGGACAATT | UUGUCCUUCAAACUUGGGCTT |
| CASP4-homo-801 | GCCUCAGUCUGAAGGACAATT | UUGUCCUUCAGACUGAGGCTT |
| ASC-homo-651 | GGCAAUCCCACCAAAUCAUTT | AUGAUUUGGUGGGAUUGCCTT |

**Supplementary Table 3. Primers sequences. Primer synthesized for qRT-PCR.**

| Gene | Sequence (5'-3') | | Product length |
| --- | --- | --- | --- |
|  | **Forward** | **Reverse** |  |
| GAPDH | GAAGGTGAAGGTCGGAGTC | GAAGATGGTGATGGGATTTC | 226 |
| IL-1β | CAGCCAATCTTCATTGCTCA | TCGGAGATTCGTAGCTGGAT | 135 |
| NLRP3 | GATCTTCGCTGCGATCAACAG | CGTGCATTATCTGAACCCCAC | 81 |
| ASC | TGGATGCTCTGTACGGGAAG | CCAGGCTGGTGTGAAACTGAA | 110 |
| Caspase-1 | GAGAAACATCCAAAAGTGAGGG | GCCTTTCTTCTGGTCAGTGC | 133 |
| Caspase-4 | CAAGAGAAGCAACGTATGGCA | AGGCAGATGGTCAAACTCTGTA | 325 |
