# Supplementary figures and images for: Porphyromonas gingivalis-Induced NLRP3 Inflammasome Activation and Its Downstream Interleukin-1β Release Depend on Caspase-4
Source: Front Microbiol. 2020 Aug 13;11:1881. doi: 10.3389/fmicb.2020.01881 (PMC7438778; doi:10.3389/fmicb.2020.01881)

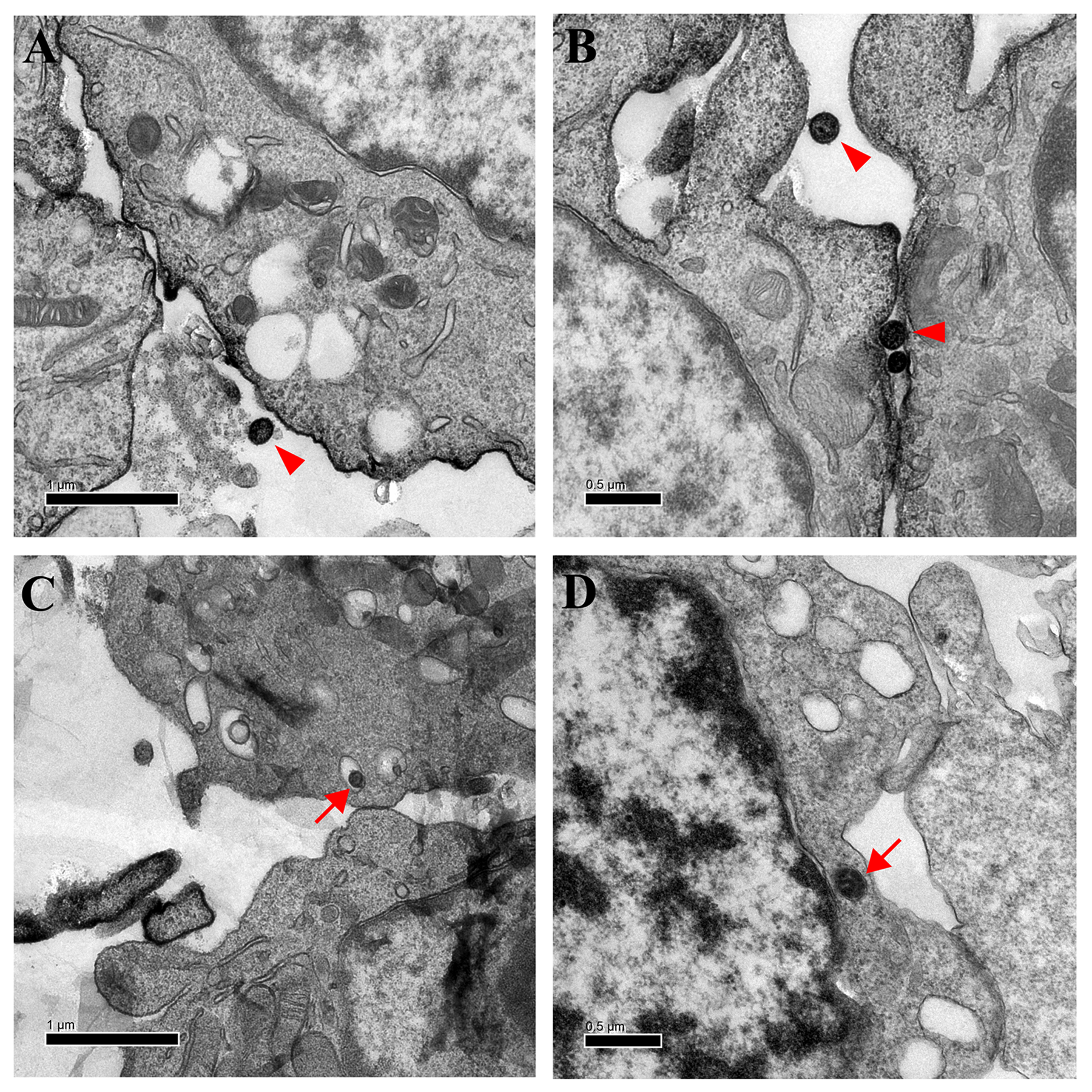

Supplement: SUPPLEMENTARY FIGURE S1 — TEM micrographs of S. mitis entry into human THP-1 cells. Transmission electron microscope images of co-culture of S. mitis ATCC 49456 (MOI = 50) and THP-1 cells after 2 h. (A) S. mitis were found outside human THP-1 cells. (B) There were several chains of S. mitis between two cells without entry. (C,D) S. mitis cells were found inside THP-1 cells, localized near the cell membrane (C) or near the nucleus (D). Arrow head: S. mitis chain outside cells; arrows: S. mitis chain inside cells. Magnification: 10,000× (A,C) and 11,500× (B,D). [file Image_1.TIF]

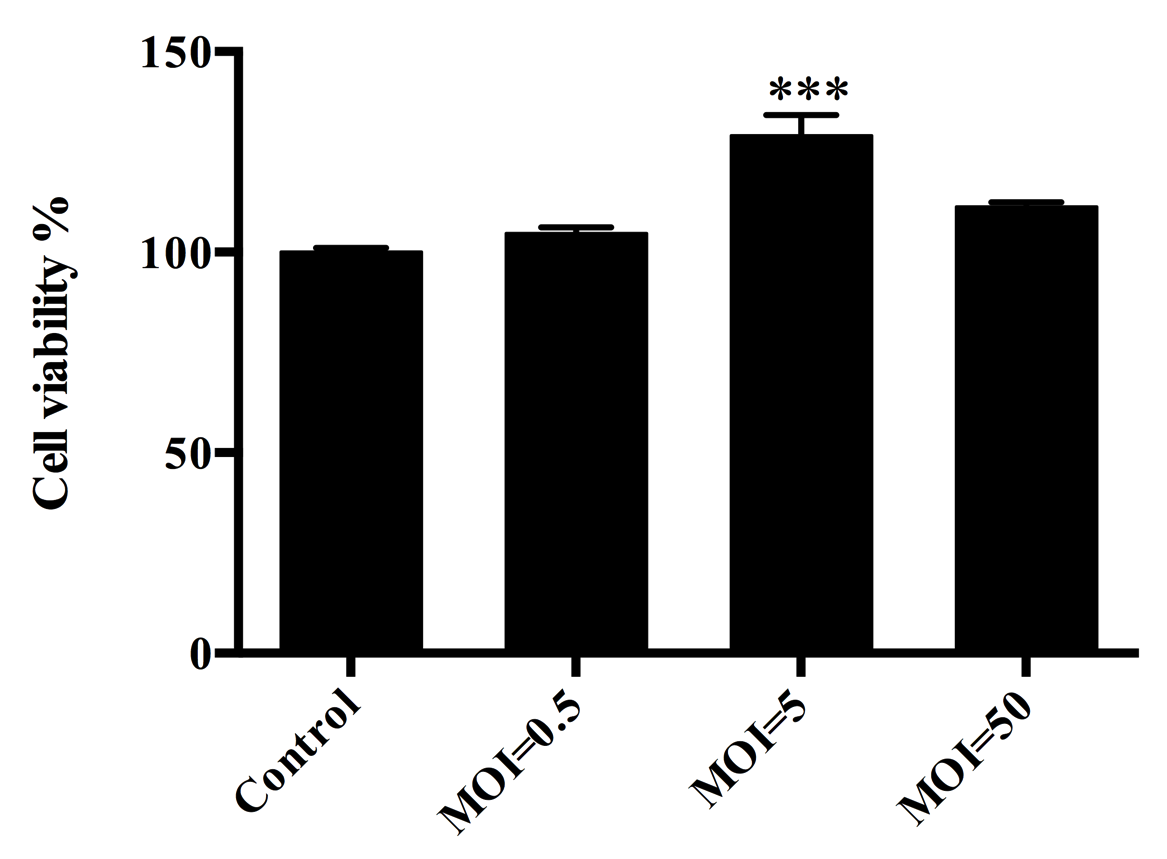

Supplement: SUPPLEMENTARY FIGURE S2 — Cell viability during P. gingivalis infection. Cell viability of human phorbol-12-myristate-13-acetate (PMA)-primed THP-1 cells was measured by CCK8 kit after P. gingivalis infection at 24 h for indicated MOIs. All doses of P. gingivalis exhibited little cell cytotoxicity after 24-h of infection. [file Image_2.TIFF]

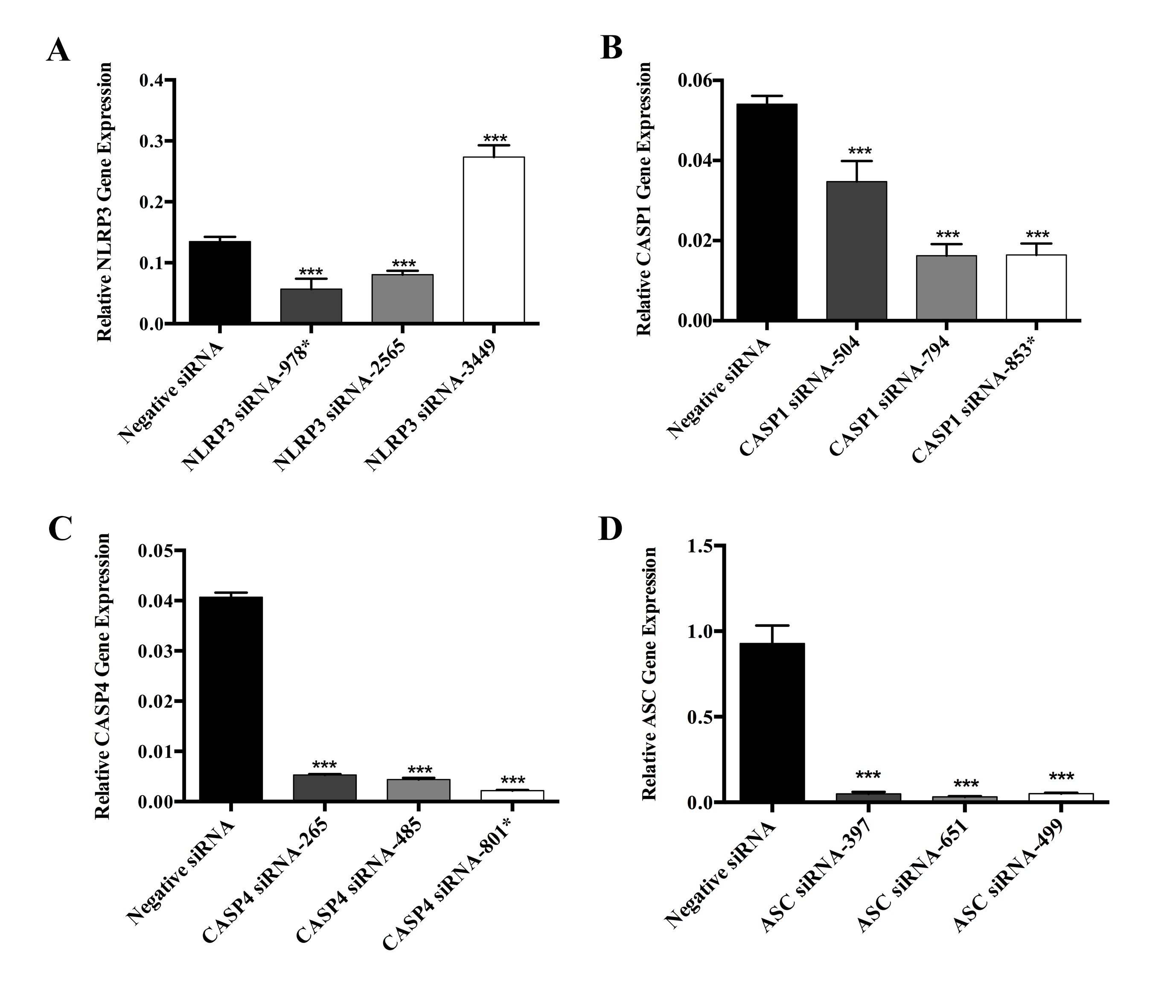

Supplement: SUPPLEMENTARY FIGURE S3 — NLRP3, CASP1, CASP4, and ASC siRNA selection and evaluation of their efficiency. PMA-primed THP-1 cells (1 × 10^6^) were transfected with three kinds of siRNA (three sequences per kind) for 24 h. Gene expression was measured by real-time qPCR. (A) NLRP3 siRNA-978 knocked down NLRP3 gene expression up to 57.8% and was selected for subsequent experiments. (B) CASP1 siRNA-853 knocked down CASP1 gene expression up to 69.6% and was selected for subsequent experiments. (C) CASP4 siRNA-801 knocked down CASP4 gene expression up to 94.6% and was selected for subsequent experiments. (D) ASC siRNA-651 knocked down ASC gene expression up to 96.6% and was selected for subsequent experiments. Data are representative of at least three experiments. *p < 0.05, **p < 0.01, and ***p < 0.001. [file Image_3.TIF]

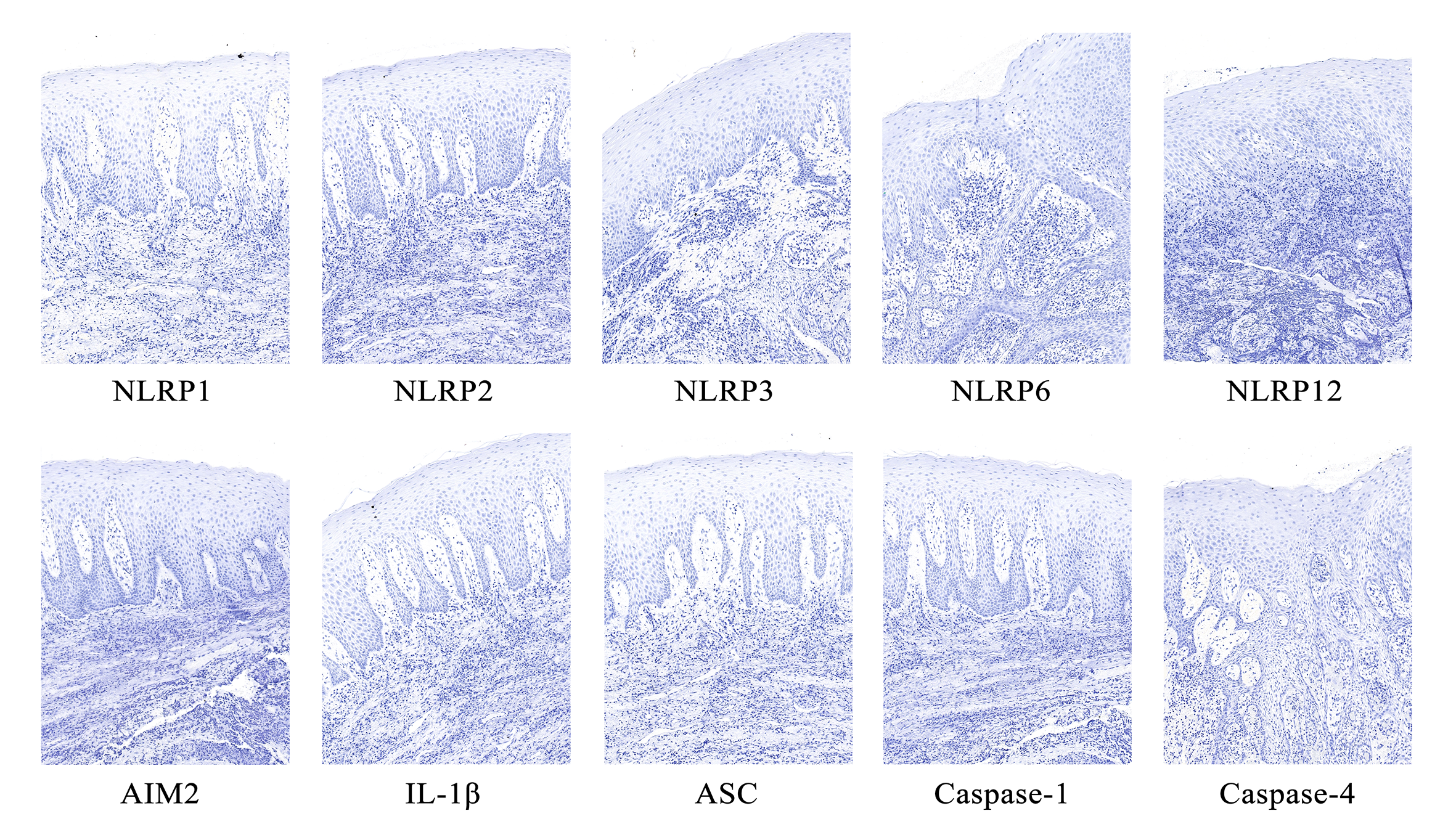

Supplement: SUPPLEMENTARY FIGURE S4 — Negative controls for NLRP1, NLRP2, NLRP3, NLRP6, NLRP12, absent in melanoma 2 (AIM2), IL-1β, ASC, Caspase-1, and Caspase-4 staining. Full-thickness gingival tissue specimens including both the epithelium and connective tissue were obtained from patients with severe chronic periodontitis. Sections were cut into 5-mm thickness and the slides were subjected to immunohistochemistry. After being washed repeatedly in phosphate-buffered saline (PBS), the slides were incubated with only the secondary antibody. Non-specific binding was not seen. Magnification: 100×. [file Image_4.TIF]

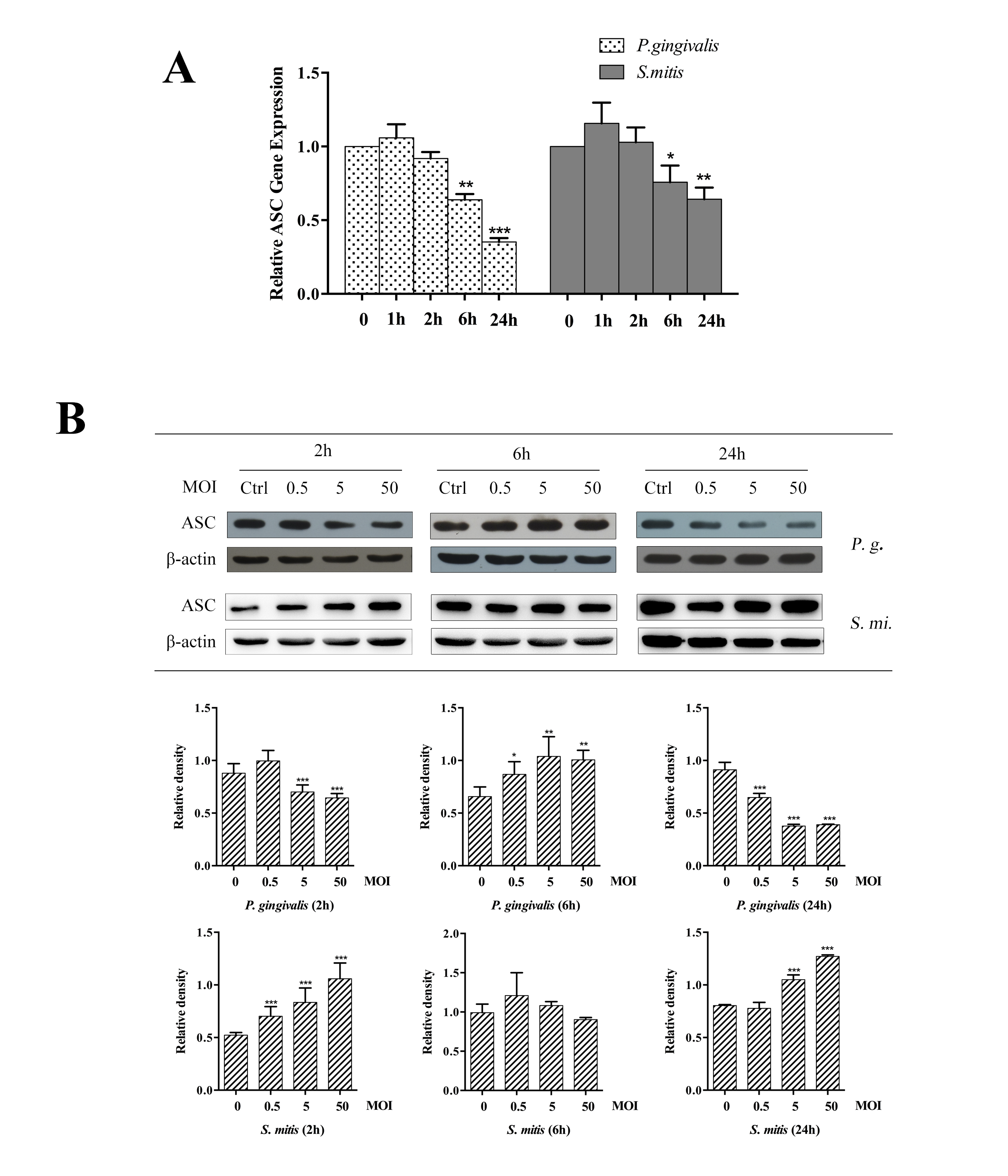

Supplement: SUPPLEMENTARY FIGURE S5 — The expression of ASC after P. gingivalis or S. mitis treatment. PMA-primed THP-1 cells were treated with P. gingivalis or S. mitis (MOI = 50) for 1, 2, 6, and 24 h. ASC mRNA expression was measured by real-time qPCR (A), ASC intracellular protein was detected by immunoblotting (B). Both P. gingivalis and S. mitis induced a slight decreasing trend in ASC mRNA expression over the experimental time course (A). In terms of protein accumulation (B), ASC significantly increased following inoculation with S. mitis at 2 h (MOI = 0.5, 5, and 50) and at 24 h (MOI = 5 and 50). However, inoculation with P. gingivalis, ASC levels significantly dropped at 2 h (MOI = 5 and 50), increased at 6 h (MOI = 5 and 50), then significantly dropped again by 24 h (MOI = 0.5, 5, and 50) of exposure (Figure 2D). The trends for ASC accumulation thus showed an inverse relationship in comparisons between THP-1 treatment with P. gingivalis and S. mitis. [file Image_5.TIF]

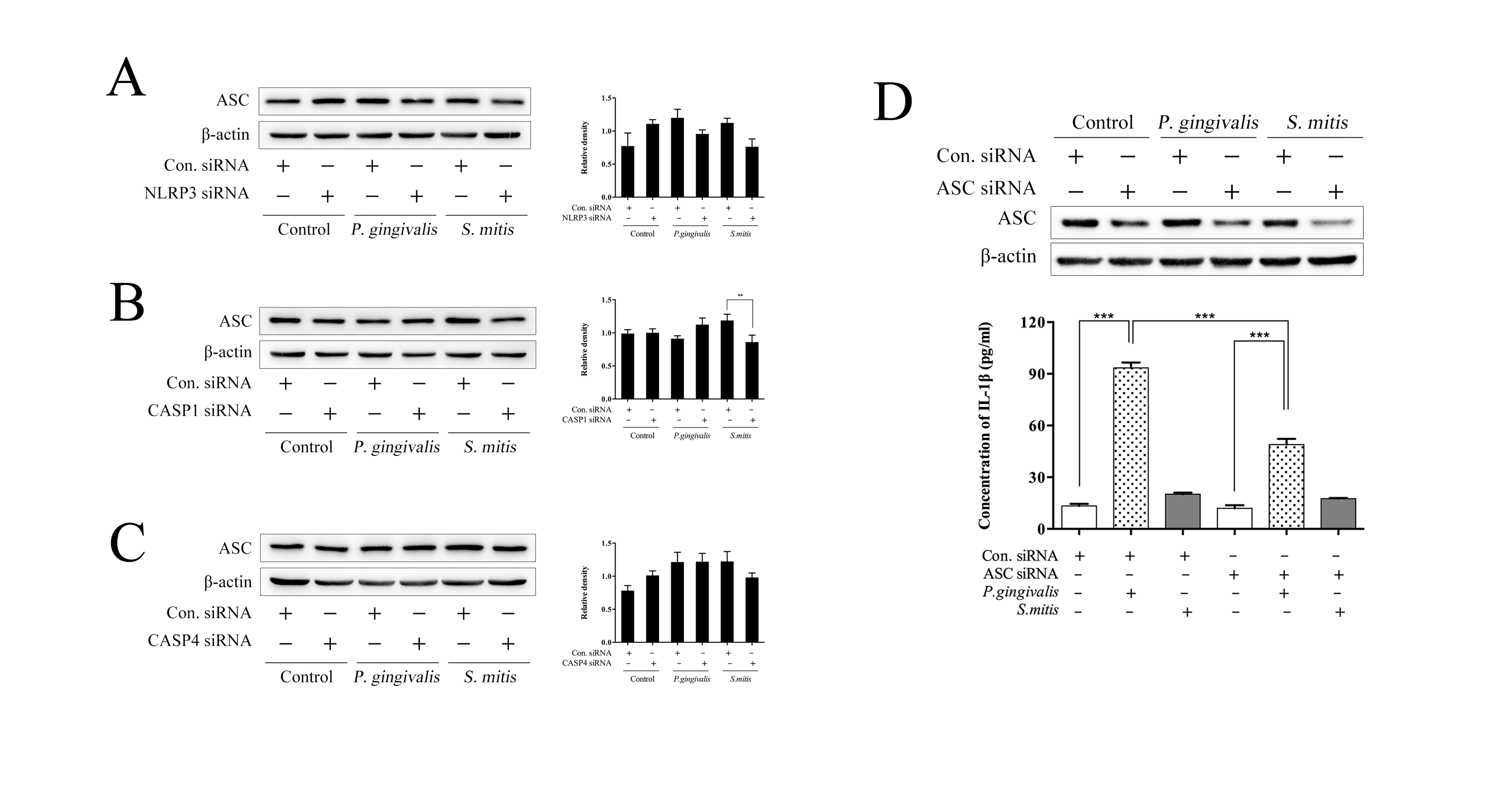

Supplement: SUPPLEMENTARY FIGURE S6 — The expression of ASC after P. gingivalis or S. mitis treatment in silenced cells. PMA-primed THP-1 cells were transfected with NLRP3, CASP1, CASP4, or ASC siRNA for 48 h. siRNA-transfected cells were treated with P. gingivalis or S. mitis (MOI = 50) for 2 h. ASC intracellular protein was detected by immunoblotting. NLRP3 or CASP4 silencing led to no significant change in ASC expression in all groups (A,C). While in CASP1 silenced cells, ASC decreased during S. mitis treatment compared to unsilenced cells (B). For ASC silencing cells, the secretion of mature IL-1β was examined by ELISA. It turned out that IL-1β was also decreased significantly when ASC silencing. [file Image_6.TIF]
